# Genome Sequences of SARS-CoV-2 Sublineage B.1.617.2 Strains from 12 Children in Chattogram, Bangladesh

**DOI:** 10.1128/MRA.00912-21

**Published:** 2021-10-21

**Authors:** Adnan Mannan, H. M. Hamidullah Mehedi, M. Abdur Rob, Sanjoy Kanti Biswas, Nahid Sultana, Rajdeep Biswas, M. Minhazul Hoque, Mohammed Akram Hossain, Ajoy Das, Kallyan Chakma, Asma Salauddin, M. Thosif Raza, Fahim Hasan Reza, Azmain Mahtab, Mojnu Miah, Rashedul Hasan, Mustafizur Rahman, Mohammed Ziaur Rahman, Mohammad Enayet Hossain

**Affiliations:** a Department of Genetic Engineering & Biotechnology, Faculty of Biological Sciences, University of Chittagong, Chattogram, Bangladesh; b Department of Medicine, 250 Bedded General Hospital, Chattogram, Bangladesh; c Chattogram Maa O Shishu Hospital, Agrabad, Chattogram, Bangladesh; d Anesthesia & Intensive Care Unit, 250 Bedded General Hospital, Chattogram, Bangladesh; e Department of Otolaryngology, 250 Bedded General Hospital, Chattogram, Bangladesh; f Department of Cardiology, 250 Bedded General Hospital, Chattogram, Bangladesh; g Department of Orthopaedics, 250 Bedded General Hospital, Chattogram, Bangladesh; h COVID-19 Screening Centre, Chevron Clinical Laboratory (Pte.) Ltd., Chattogram, Bangladesh; i Virology Laboratory, International Centre for Diarrhoeal Disease Research, Bangladesh (icddr,b), Dhaka, Bangladesh; DOE Joint Genome Institute

## Abstract

We announce the complete genome sequences of 12 severe acute respiratory syndrome coronavirus 2 (SARS-CoV-2) sublineage B.1.617.2 strains (Delta variant) obtained from nasopharyngeal and oropharyngeal swab samples from 12 pediatric patients in Chittagong, Bangladesh, displaying COVID-19 symptoms. Oxford Nanopore MinION sequencing technology was used to generate the genomic sequences.

## ANNOUNCEMENT

Severe acute respiratory syndrome coronavirus 2 (SARS-CoV-2) is a single-stranded RNA virus that belongs to the genus *Betacoronavirus* of the family *Coronaviridae*. This causative agent of coronavirus disease 2019 was first reported in Bangladesh on 8 March 2020. The complete sequences of 12 SARS-CoV-2 isolates from 12 children are presented in this paper.

As part of the countrywide COVID-19 laboratory network, the International Centre for Diarrhoeal Disease Research, Bangladesh (icddr,b), 250 Bedded General Hospital, Chattogram, and the Chattogram Maa O Shishu Hospital have been testing for SARS-CoV-2 since April 2020. Due to the recent emergence of the SARS-CoV-2 delta variant in Bangladesh, we wanted to evaluate its presence in COVID-19-positive children. Between 1 June 2021 and 7 July 2021, we enrolled 12 children with symptoms of COVID-19 and collected nasopharyngeal and oropharyngeal swab samples (*n* = 12). The presence of SARS-CoV-2 was determined by real-time reverse transcriptase PCR using ORF1ab- and N gene-specific primers/probes (1).

The QIAamp viral RNA minikit (Qiagen) was used for extraction of the viral RNA. Sequencing libraries were prepared following the nCoV-2019 sequencing protocol v3 (LoCost) (2). Briefly, a batch of 24 amplified samples was barcoded prior to being pooled for purification and sequencing adapter ligation. The final libraries were quantified and loaded onto a FLO-MIN106D (R9.4.1) flow cell on an Oxford Nanopore MinION MK 1C platform for 8 h. In total, 2,890,603 reads (range, 71,821 to 170,241 reads per sample; average length, 499 bp) were generated by real-time base calling using Guppy 4.3.4 as released with MinKNOW software in fast base-calling mode. A consensus FASTA file was generated using a FASTQ QC + ARTIC + Nextclade r1.0.4 workflow on the cloud-based analysis platform EPI2ME Desktop Agent v3.3.0 with default parameters, based on ARTIC Field Bioinformatics software (https://github.com/artic-network/fieldbioinformatics). Consensus genome sequences were achieved with 99.55% coverage at a minimum coverage of 10× and maximum of 400×.

**FIG 1:**
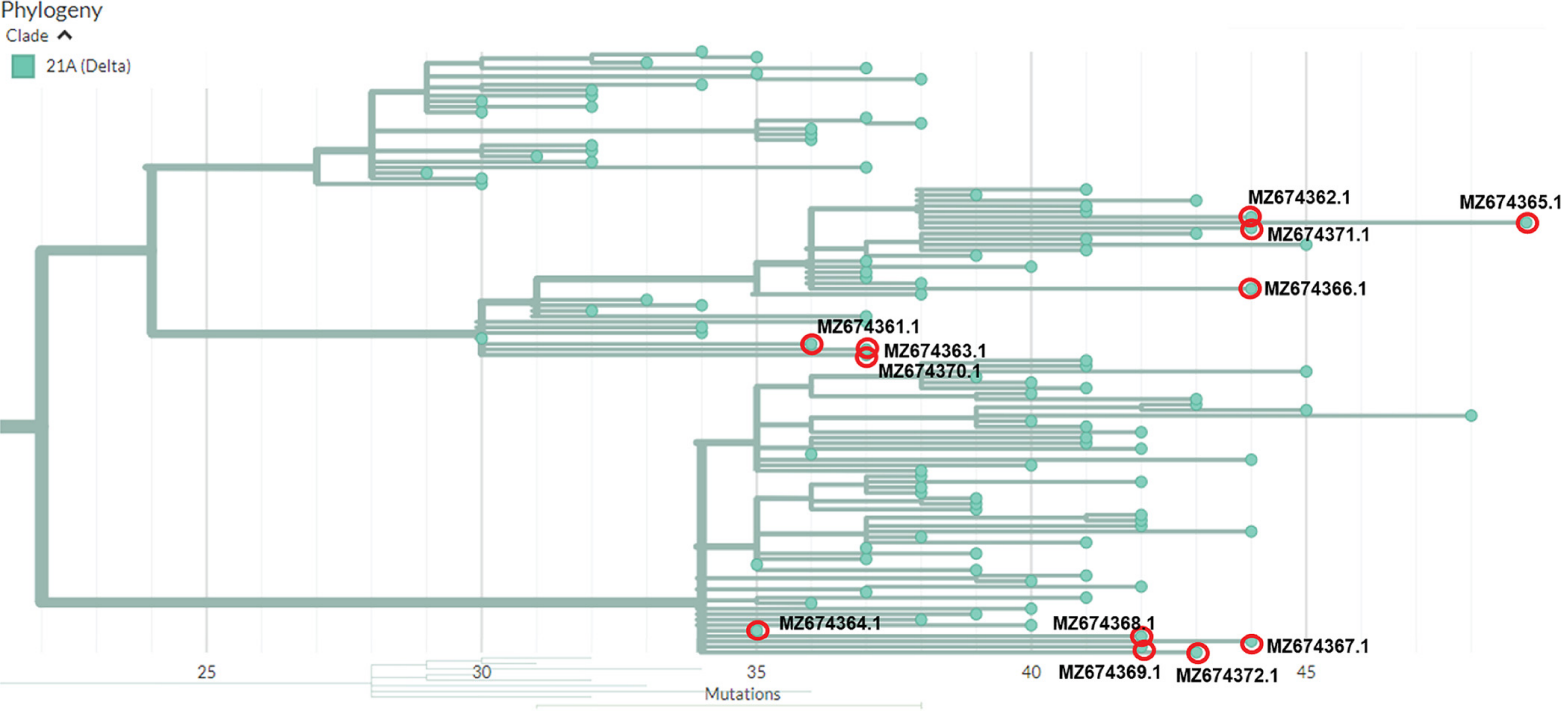
Phylogenetic tree of the 12 SARS-CoV-2 isolates from Chittagong, Bangladesh. The *x* axis represents the number of mutations from the SARS-CoV-2 Wuhan Hu-1 reference genome. The red circles indicate the 12 isolates that have fallen under the region of clade 21A (Delta).

A phylogenetic tree was constructed on 3 August 2021 using the Nextclade v1.5.2 (https://clades.nextstrain.org/) with default parameters (3). All 12 of the genome sequences branched in clade 21A (Fig. 1). According to the GISAID database Basic Local Alignment Search Tool (BLAST) (4), the genomes share the highest levels of similarity with sequences uncovered from the Indian delta variant (GISAID accession number EPI_ISL_2029113). According to analysis using Phylogenetic Assignment of Named Global Outbreak Lineages (Pangolin) with default parameters (https://pangolin.cog-uk.io/) (5), 4 of the 12 sequences are reported to be from delta sublineage AY.4, while the rest are from lineage B.1.617.2.

The availability of genomic data for circulating SARS-CoV-2 strains from children of different parts of the world will serve as a valuable resource for monitoring the infection patterns of different variants among children. Overall, it will help with infection control and management of SARS-CoV-2 spread among children.

### Data availability.

The data from this study can be found at NCBI under the BioProject accession number PRJNA766295. The complete nucleotide sequences of these SARS-CoV-2 strains have been deposited in the GISAID database under the accession numbers EPI_ISL_2987440, EPI_ISL_2987441, EPI_ISL_2987442, EPI_ISL_2987443, EPI_ISL_2987444, EPI_ISL_2987445, EPI_ISL_2987446, EPI_ISL_2987447, EPI_ISL_2987448, EPI_ISL_2987449, EPI_ISL_2987450, and EPI_ISL_2987451. The Sequence Read Archive (SRA) and GenBank accession numbers are listed in Table 1.

**TABLE 1:** Mutations observed in our sequences compared with SARS-CoV-2 isolate Wuhan-Hu-1

GenBank accession no.	SRA accession no.	Genome size (bp)	Coverage (%)	GC content (%)	Nucleotide variations	Amino acid variations
MZ674361	SRR16071953	29,769	99.55	37.99	G210T, C241T, C1191T, C1267T, C3037T, G3875A, C5184T, C6539T, C9891T, T11418C, T12946C, C14262T, C14408T, G15451A, T16326C, C16466T, C17288T, A20262G, C20320T, C21618G	ORF1ab: P129L, P822L, D70G, A446V, V149A, P323L, G671S, P77L, P46L, A108V; S protein: T19R, E156G, F157del, R158del, L452R, T478K, D614G, P681R, D950N, L1141W; NS3: S26L; E protein: V62F; M protein: I82T; NS6: W27L; NS7a: V82A, L116F, T120I; N protein: D63G, R203M, D377Y
MZ674362	SRR16071952	29,769	99.55	37.98	G210T, C241T, C1191T, C1267T, C3037T, G3875A, C5184T, C6539T, C9891T, T11418C, T12946C, C14262T, C14408T, G15451A, T16326C, C16466T, C17288T, A20262G, C20320T, C21618G	ORF1ab: P129L, A386T, P822L, H1274Y, A446V, V149A, P323L, G671S, P77L, T351I, H234Y; S protein: T19R, E156G, F157del, R158del, W258L, L452R, T478K, D614G, P681R, D950N; NS3: S26L; M protein: I82T
MZ674363	SRR16071949	29,769	99.55	37.99	G210T, C241T, C1191T, C1267T, C3037T, C5144T, C5184T, C9561T, C9891T, T11418C, T12946C, C14408T, C15240T, G15451A, C16466T, C18176T, A20262G, C21618G, T22917G, C22995A	ORF1ab: P129L, P822L, S336L, A446V, V149A, P323L, G671S, P77L, P46L; S protein: T19R, E156G, F157del, R158del, L452R, T478K, D614G, P681R, D950N, L1141W; NS3: S26L; E protein: V62F; M protein: I82T
MZ674364	SRR16071948	29,769	99.55	38	G210T, C241T, C3037T, G4181T, C6402T, C7124T, C8986T, G9053T, C10029T, A11201G, A11332G, C14408T, G15451A, C16466T, C19220T, A20130T, C21618G, T22917G, C22995A, A23403G	ORF1ab: A488S, P1228L, P1469S, V167L, T492I, T77A, P323L, G671S, P77L, A394V, E170D; S protein: T19R, E156G, F157del, R158del, L452R, T478K, D614G, P681R, D950N; NS3: S26L; M protein: I82T; NS7a: V82A, T120I; NS7b: T40I; N protein: D63G, R203M, G215C, D377Y
MZ674365	SRR16071947	29,769	99.55	37.99	G210T, C241T, Cl19IT, C1267T, T1623C, C3037T, G5012T, C5184T, C6539T, C7420T, C9891T, T11418C, T12946C, C13430T, C14262T, C14408T, G15451A, T16050C, C16466T, A17207G	ORF1ab: P129L, I273T, V765F, P822L, H1274Y, A446V, V149A, P136S, P323L, G671S, P77L, Y324C, H234Y; S protein: T19R, E156G, F157del, R158del, L452R, T478K, D614G, P681R, D950N; NS3: I20T, S26L, V225F; M protein: I82T; NS7a: V82A, L116F, T120I; N protein: D63G, R203M, T362I, D377Y, R385K
MZ674366	SRR16071946	29,769	99.55	37.97	G210T, C241T, C1191T, C1267T, C3037T, C5184T, C6539T, G7850T, C9891T, T11418C, G12067T, C12786T, T12946C, C14262T, C14408T, G15451A, C16466T, C19972T, A20262G, C20320T	ORF1ab: P129L, P822L, H1274Y, A1711S, A446V, V149A, M75I, T34I, P323L, G671S, P77L, P118S, H234Y; S protein: T19R, E156G, F157del, R158del, L452R, T478K, D614G, P681R, D950N; NS3: S26L; M protein: I82T; NS7a: V82A, L116F, T120I
MZ674367	SRR16071945	29,769	99.55	37.95	G210T, C241T, C2910T, C3037T, C3743T, C3787T, C4181T, C6402T, C7124T, G7936C, T8365C, C8986T, G9053T, C10029T, G11083T, A11201G, A11332G, C13368T, C14408T, G15451A	ORF1ab: T64I, H342Y, A488S, P1228L, P1469S, V167L, T492I, L37F, T77A, T115I, P323L, G671S, P77L, A394V, S288F; S protein: T19R, G142D, E156G, F157del, R158del, L452R, T478K, D614G, P681R, D950N; NS3: S26L; M protein: I82T; NS7a: V82A, T120I; NS7b: T40I; N protein: D63G, R203M, G215C, D377Y
MZ674368	SRR16071944	29,769	99.55	37.98	G210T, C241T, C2910T, C3037T, C3743T, C3787T, C4181T, C6402T, C7124T, G7936C, T8365C, C8986T, G9053T, C10029T, A11201G, A11332G, C13368T, C14408T, G15451A, C16466T	ORF1ab: T64I, H342Y, A488S, P1228L, P1469S, V167L, T492I, T77A, T115I, P323L, G671S, P77L, A394V; S protein: T19R, E156G, F157del, R158del, L452R, T478K, D614G, P681R, D950N; NS3: S26L; M protein: I82T; NS7a: V82A, T120I; NS7b: T40I
MZ674369	SRR16071943	29,769	99.55	37.99	G210T, C241T, C2910T, C3037T, C3743T, C3787T, C4181T, C6402T, C7124T, G7936C, T8365C, C8986T, G9053T, C10029T, A11201G, A11332G, C13368T, C14408T, G15451A, C16466T	ORF1ab: T64I, H342Y, A488S, P1228L, P1469S, V167L, T492I, T77A, T115I, P323L, G671S, P77L, A394V; S protein: T19R, E156G, F157del, R158del, L452R, T478K, D614G, P681R, D950N; NS3: S26L; M protein: I82T
MZ674370	SRR16071942	29,769	99.55	37.99	G210T, C241T, C1191T, C1267T, C3037T, A3469C, G4985A, C5184T, C8956T, C9891T, T11418C, T12946C, C14408T, A15063G, G15451A, C16466T, C18716T, A20262G, C21618G, T22917G	ORF1ab: P129L, V756I, P822L, A446V, V149A, P323L, G671S, P77L, P46L; S protein: T19R, E156G, F157del, R158del, L452R, T478K, D614G, P681R, D950N, C1243F; NS3: S26L; M protein: I82T; NS7a: V82A, L116F, T120I; N protein: D63G, R203M, D377Y
MZ674372	SRR16071951	29,769	99.55	37.98	G210T, C241T, C593T, A1395G, C3037T, G4181T, C5806T, C6402T, A6613G, C7124T, C8986T, G9053T, C10029T, A11201G, A11332G, C14408T, G15451A, C16466T, C19220T, C21575T	ORF1ab: H110Y, E197G, A488S, P1228L, P1469S, V167L, T492I, T77A, P323L, G671S, P77L, A394V; S protein: L5F, T19R, E156G, F157del, R158del, L452R, T478K, D614G, P681R, D950N; NS3: K21R, S26L; M protein: I82T; NS6: P57L; NS7a: V82A, T120I; NS7b: T40I; NS8: V114I; N protein: D63G, R203M, G215C, D377Y
MZ674371	SRR16071950	29,769	99.55	37.99	G210T, C241T, C1191T, C1267T, C3037T, G3047T, G3875A, C5184T, C6539T, T8918T, C9891T, T11418C, T12946C, C14262T, C14408T, G15451A, T16326C, C16466T, A20262G, C20320T	ORF1ab: P129L, D110Y, A386T, P822L, H1274Y, A446V, V149A, P323L, G671S, P77L, H234Y; S protein: T19R, E156G, F157del, R158del, L452R, T478K, D614G, P681R, D950N, S1252F; NS3: S26L; M protein: I82T; NS7a: V82A, L116F, T120I; N protein: D63G, R203M, T362I, D377Y, R385K

### Ethical approval.

This study has been approved by the research review board of Chattogram Maa O Shishu Hospital Medical College (protocol number CMOSHMC/IRB/2021/17).
